# Differences in Whole Blood Gene Expression Associated with Infection Time-Course and Extent of Fetal Mortality in a Reproductive Model of Type 2 Porcine Reproductive and Respiratory Syndrome Virus (PRRSV) Infection

**DOI:** 10.1371/journal.pone.0153615

**Published:** 2016-04-19

**Authors:** Jamie M. Wilkinson, Andrea Ladinig, Hua Bao, Arun Kommadath, Paul Stothard, Joan K. Lunney, John C. S. Harding, Graham S. Plastow

**Affiliations:** 1 Department of Agricultural, Food, and Nutritional Science, University of Alberta, Edmonton, AB, Canada; 2 Department of Large Animal Clinical Sciences, Western College of Veterinary Medicine, University of Saskatchewan, Saskatoon, SK, Canada; 3 Animal Parasitic Diseases Laboratory, Beltsville Agricultural Research Center, Agricultural Research Service, U.S. Department of Agriculture, Beltsville, Maryland, United States of America; Washington State University, UNITED STATES

## Abstract

Porcine Reproductive and Respiratory Syndrome Virus (PRRSV) infection of pregnant females causes fetal death and increased piglet mortality, but there is substantial variation in the extent of reproductive pathology between individual dams. This study used RNA-sequencing to characterize the whole blood transcriptional response to type 2 PRRSV in pregnant gilts during the first week of infection (at 0, 2, and 6 days post-inoculation), and attempted to identify gene expression signatures associated with a low or high level of fetal mortality rates (LFM and HFM; n = 8/group) at necropsy, 21 days post-inoculation. The initial response to infection measured at 2 days post-inoculation saw an upregulation of genes involved in innate immunity, such as interferon-stimulated antiviral genes and inflammatory markers, and apoptosis. A concomitant decrease in expression of protein synthesis and T lymphocyte markers was observed. By day 6 the pattern had reversed, with a drop in innate immune signaling and an increase in the expression of genes involved in cell division and T cell signaling. Differentially expressed genes (DEGs) associated with extremes of litter mortality rate were identified at all three time-points. Among the 15 DEGs upregulated in LFM gilts on all three days were several genes involved in platelet function, including integrins *ITGA2B* and *ITGB3*, and the chemokine *PF4 (CXCL4)*. LFM gilts exhibited a higher baseline expression of interferon-stimulated and pro-inflammatory genes prior to infection, and of T cell markers two days post-infection, indicative of a more rapid progression of the immune response to PRRSV. This study has increased our knowledge of the early response to PRRSV in the blood of pregnant gilts, and could ultimately lead to the development of a biomarker panel that can be used to predict PRRSV-associated reproductive pathology.

## Introduction

Porcine Reproductive and Respiratory Syndrome (PRRS), caused by the Porcine Reproductive and Respiratory Syndrome Virus (PRRSV), poses a serious animal health and economic challenge to pig producers worldwide. In juvenile swine, PRRS is characterized by respiratory disease, slow growth, and high mortality. In gilts and sows, PRRSV infection in late gestation is characterized by abortions and an increase in the proportion of dead and weak-born piglets in the litter at farrowing [1]. PRRS vaccines are available, but do not provide complete protection against heterologous viral strains [2], and so complementary disease prevention strategies are sought.

The severity of reproductive disease varies considerably. Differences in viral strains and management practices are important contributory factors, but it is evident from previous studies of PRRS outbreaks on individual farms that sow genetics also contributes to this variation [3, 4]. This opens up the possibility of selecting replacement gilts for pig herds that are either resistant or tolerant to PRRS, which would be of great benefit to the pig industry. We recently undertook the largest experimental inoculation study to date to investigate the reproductive form of PRRS in late gestation pregnant gilts and their fetuses. In this study, no gilts exhibited complete resistance to PRRSV infection. All 111 inoculated gilts became viremic post-inoculation, but there was significant variation in litter pathology, with the percentage (%) of fetuses within a litter that contained a detectable amount of virus ranging from 0% to 100%, and fetal mortality rate ranging from 0% to 94% [5]. Several gilt- and fetal-level phenotypic factors were found to be associated with severity of reproductive PRRS in this experimental model [6]. At the gilt-level, these phenotypic factors included cytokine responses in serum and supernatants of *ex vivo* PRRSV-stimulated peripheral blood mononuclear cells (PBMC), and absolute levels of different leukocyte subsets.

Transcriptional responses during the early innate immune response to infection can have a large effect on subsequent disease outcomes. In a growing pig model of PRRS for example, differences in blood transcriptional response of individual animals during the first week of infection were found to correlate with weight gain over a 42-day period, a phenotypic measure of tolerance to infection [7]. Others have identified expression signatures by which the transcript levels of a relatively small number of genes at an early point post-infection can be used to reliably predict subsequent clinical outcomes following influenza and dengue virus infection in humans [8, 9]. The specific aims of this study were two-fold: firstly, to characterize the transcriptomic response to infection in gilt blood during the first six days of infection, and secondly, to search for gene expression signatures in gilt blood that are associated with severity of litter pathology.

## Materials and Methods

### Ethics statement

Inoculation of pregnant gilts or sows with PRRSV during late gestation is the only effective method of studying the effects of infection in the maternal and fetal compartments of the reproductive tract. A humane intervention point (HIP) checklist was developed for this project to ameliorate animal suffering. Gilts were monitored twice daily according to the HIP checklist, but clinical signs in gilts were mild or absent, meaning that medical treatments such as analgesics and anesthetics were not required. PRRSV inoculation of pregnant gilts results in the death of some fetuses, and this was the case for this study. It is impossible to predict if and when an individual fetus will die following gilt inoculation, and monitoring fetuses for signs of stress is not feasible in a litter bearing species like the pig. Indeed, the extent of fetal mortality was an important variable used for selecting the animals used in this study. Given that fetal death was an outcome of this study, the experimental protocol was carefully considered by the University of Saskatchewan’s Animal Research Ethics Board before they approved it. The protocol adhered to the Canadian Council of Animal Care guidelines for humane animal use (protocol #20110102).

### PRRSV challenge and sampling procedures

A detailed description of the animal experiment is available in Ladinig *et al* [5]. Briefly, 114 pregnant (gestation day 85±1) Landrace gilts, confirmed seronegative for PRRSV, were inoculated with 1x10^5^ TCID_50_ type 2 PRRSV isolate NVSL 97–7895 on experimental day 0 (D0). Blood samples for transcriptomic analyses were collected into Tempus blood RNA tubes (Thermo Fisher Scientific, Waltham, MA, USA) on D0, D2, and D6 post-inoculation. Heparinized blood samples for flow cytometry [10] and serum samples for PRRSV quantification and cytokine analysis [11] were also collected. Gilts were humanely euthanized and necropsied at D21 (gestation day 106±1). The preservation status of each fetus in the gilt uterus was recorded, and the fetal mortality rate was calculated for each litter. PRRSV RNA concentrations in gilt blood and fetal thymus were determined by quantitative reverse transcription polymerase chain reaction as described previously [5].

### RNA isolation and sequencing

Transcriptomic analyses of whole blood were performed on a subset of 16 animals selected from the 114 PRRSV-challenged gilts. This subset consisted of two groups of gilts whose litters exhibited divergent levels of fetal pathology and PRRSV RNA concentrations. The low fetal mortality (LFM) group (n = 8) had a mean fetal mortality rate of 5% (range 0–13%) and a mean fetal thymus PRRSV titer of 0.74 log_10_ RNA copies/mg (range 0–1.87). The high fetal mortality (HFM) group (n = 8) had a mean fetal mortality rate of 76% (range 67–94%) and a mean fetal thymus PRRSV titer of 4.43 log_10_ RNA copies/mg respectively (range 3.66–5.62) (S1 Fig). Only gilts with a minimum litter size of 8 were selected, and there was no significant difference in the mean litter size of the HFM and LFM groups (13.9 and 15.0 respectively). Total RNA was isolated from each of the gilt blood samples collected on D0, D2, and D6, using the Preserved Blood RNA Purification kit (Norgen Biotek, Thorold, ON, Canada). The highly abundant *HBA1*, *HBA2*, and *HBB* globin transcripts were selectively depleted from each RNA sample according to a previously published method [12] in order to increase the sensitivity of RNA-sequencing for detection of rare transcripts and differential expression. RNA was quantified by spectrophotometry using a Nanodrop ND 2000 (Thermo Fisher Scientific). RNA quality for library construction was assessed by digital electrophoresis using a 2200 Tapestation (Agilent Technologies, Santa Clara, USA). The mean sample RNA Integrity Number (RIN) was 7.9 (range 7.1–9.3). Mean RIN values were not significantly different between LFM and HFM groups or time-points (D0, D2, and D6).

Individual libraries (n = 48) for sequencing were constructed using 1 μg total RNA, and the TruSeq RNA sample preparation kit v2 (Illumina, San Diego, USA), according to the manufacturer’s instructions. Paired-end sequencing (100 base pairs) was performed on a HiSeq 2000 machine with 8 libraries per flow cell lane (Illumina), producing a mean read depth of approximately 25 million paired-end reads per sample. Reads were removed that were either flagged as low quality by CASAVA 1.8 (Illumina), had a mean PHRED quality score below 15, or for which ≥5 of the last 10 bases had a quality score below 2. Reads were aligned to the pig reference genome sequence assembly (Sscrofa10.2) using TopHat 1.4.0 with default parameters [13]. The gene annotation information used for Sscrofa10.2 was from Ensembl 71 [14]. The number of reads uniquely mapped to each gene was determined using Ht-seq count (v0.5.3.p3) [15].

### Transcriptomic and functional annotation analyses

Differentially expressed genes (DEG) were identified using the Bioconductor package ‘edgeR’ [16]. Genes with very low expression levels (CPM<1 in at least 8 samples) were filtered out of the dataset. Expression data were then normalized using the trimmed mean of M values method to adjust for any differences in RNA composition and library size [17]. A negative binomial model was fitted to the data and gene-wise dispersions were estimated using the quantile-adjusted conditional maximum likelihood method [18]. Differential gene expression was assessed using an exact test between time-points (D2 v D0 and D6 v D2) and between groups at each time-point (LFM v HFM). For the between time-point contrasts, genes were classified as differentially expressed if they had an absolute fold change >1.5 and a Benjamini-Hochberg (B-H) adjusted *P* value <0.05 [19]. For the between group contrasts, less stringent cut-offs for differential expression were applied (1.2 fold change and unadjusted *P*<0.05). An additional Gene Set Enrichment Analysis method was conducted on the LFM v HFM expression data to generate more statistically robust results at the level of the gene set as opposed to individual genes (see below). RNA-sequence and expression data are available in the NCBI Geo database under the identifier GSE75304.

Clustering of samples was performed by multidimensional scaling in two dimensions using the plotMDS function in the bioconductor package ‘limma’ [20]. Samples were clustered according to the Euclidian distance between samples pairs for the 500 genes with the largest standard deviation between samples.

Functional annotation of the LFM v HFM gene expression data was performed using Gene Set Enrichment Analysis (GSEA) [21]. Functional annotation of the DEG lists from the time-point experiments were carried out using the Ingenuity Pathway Analysis program (IPA, Qiagen Redwood City, www.qiagen.com/ingenuity). The human orthologs of the porcine genes in the analyses were obtained from the Ensembl database using the BioMart web interface [22]. GSEA was run on pre-ranked lists of all genes from the edgeR analyses. Ranking was by statistical significance and direction of expression with the trait of interest (i.e. positive log2 fold change values at the top of the list and negative values at the bottom). Gene sets that were significantly associated with day post-infection or fetal mortality groups were identified from the Hallmark collection of gene sets in the Molecular Signatures Database v5.1 [21]. Gene set enrichment scores were calculated using the ‘classic’ method, recommended for RNA-seq data analysis on the GSEA website (http://software.broadinstitute.org/gsea/index.jsp). The null distributions used to calculate the statistical significance of the enrichment scores were generated by permutation of gene sets for each contrast. Adjustment for multiple hypotheses testing was performed by determination of False Discovery Rate (FDR) [19]. Enrichment scores with a FDR <0.25 were considered to be significant.

For IPA, gene IDs and log_2_ fold changes for DEGs were imported and mapped to their corresponding objects in the Ingenuity Knowledge Database (IKB) (IPA, Qiagen Redwood City, www.qiagen.com/ingenuity). Separate lists of up and down regulated genes were analyzed using Canonical Pathway analysis. Analyses used a right-tailed Fisher’s Exact Test to identify pathways that were enriched in the gene set compared to the reference set (all genes in the human genome). A B-H adjusted *P*<0.01 cutoff and a minimum of 5 DEGs mapped to the pathway were requirements for statistical significance.

## Results and Discussion

### Temporal changes in whole blood gene expression in PRRSV-infected gilts

A total of 1954 genes were identified as differentially expressed between D2 and D0 post-inoculation from all gilts (n = 16). Between D6 and D2, 1592 genes were differentially expressed. A total of 1098 genes were differentially expressed in both contrasts, with almost all genes (1091) increasing in expression on D2 and decreasing on D6 (909) or vice-versa (182) (S1 Appendix). With regard to functional annotation, 84 pathways were enriched in DEGs from the D2 v D0 contrast (80 from the upregulated genes at D2; 4 from the downregulated genes at D2) and 105 pathways were enriched in DEGs from the D6 v D2 contrast (17 in the upregulated genes at D6; 88 in the downregulated genes at D6) (S2 Appendix).

**Fig 1:**
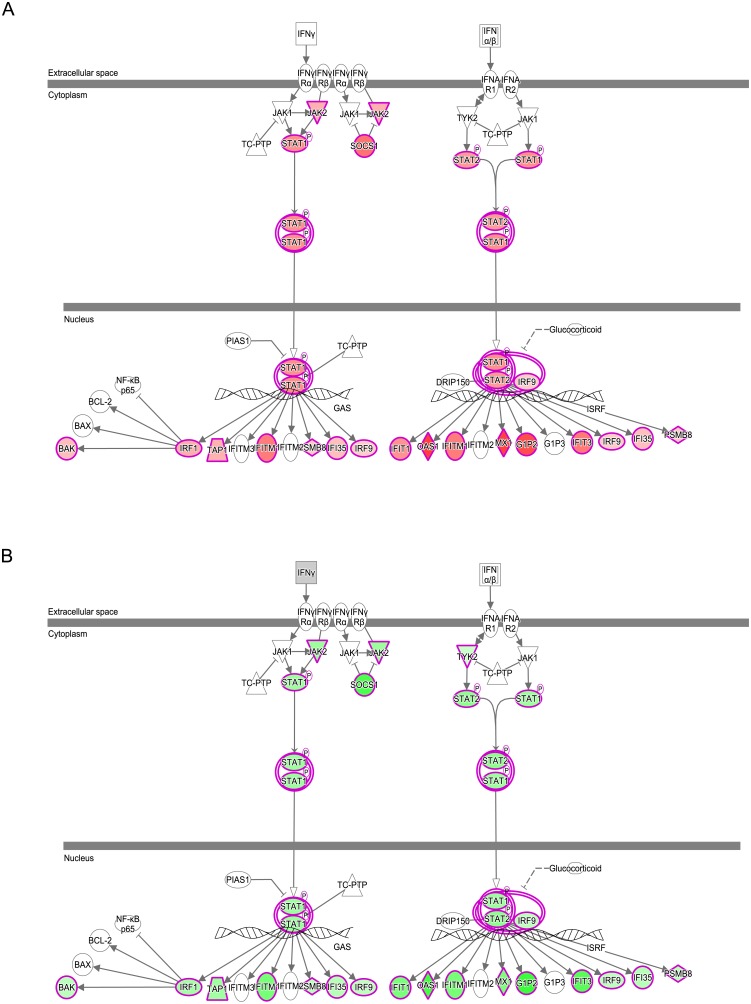
Interferon Signaling in Gilt Blood following PRRSV Infection. Type I and II interferon signaling pathways from Ingenuity Pathway Analysis. Genes whose expression is upregulated (red) at D2 compared to D0 (A) were also found to be downregulated (green) at D6 compared to D2 (B). Color intensity indicates magnitude of differential expression.

Interferon signaling was the most significant pathway upregulated at D2 compared to D0. It was also one of the most significant pathways to be downregulated at D6 compared to D2. Many of the DEGs that are activated by, or regulate interferon signaling, are common to type I and II signaling pathways but some genes that are specific to type I or type II signaling were also identified, an indication that both pathways are likely involved in the initial response to PRRSV inoculation. DEGs that are involved or regulated by type I (principally interferon alpha or beta) and/or type II (interferon gamma) interferon signaling, and map to the IPA pathways, include the transcription factors *IRF1*, *IRF9*, *STAT1* and *STAT2*, other signal transduction enzymes (*JAK2*, *SOCS1*), and a variety of molecules that interfere with virus replication within the cell (*IFIT1*, *IFITM1*, *MX1*, and *OAS1*) (Fig 1).

Interferon signaling is a component of the innate immune system, and is crucial to the control of viral infections through its inhibition of viral replication [23]. The increase in interferon signaling at D2, and its reduction at D6, mirrored the changes in IFN-α and IFN-γ proteins observed in serum from the complete set of gilts from this challenge model [11]. An increase in the *in vivo* expression of interferon-regulated genes in tissues of PRRSV-infected pigs has also been observed in other transcriptomic analyses [7, 24]. The drop off in signaling between D2 and D6 could be due to negative feedback of signal transduction or immune evasion by the virus. PRRSV employs a variety of strategies to disrupt interferon-signaling pathways, which is key to its immunosuppressive capabilities and persistent course of infection [25].

The most significant pathway associated with genes downregulated at D2 v D0 was Eukaryotic translation Initiation Factor 2 (eIF2) signaling. EIF2 is required for the initiation of cellular protein synthesis, and 40 of the DEGs in this pathway encode for ribosomal subunit proteins (*RPL13A*, *RPL32*, *RPS5*, *RPS20*, etc). The coincident increase in interferon signaling at the same time-point may be directly linked to this phenomenon. Inhibition of cellular protein synthesis is one of the hallmarks of interferon signaling [26], and the gene for the interferon-inducible kinase that negatively regulates eIF2, *EIF2AK2*/*PKR*, was upregulated in the blood of gilts at D2. Other protein synthesis pathways were also found to be significantly downregulated, including the ‘Regulation of eIF4 and p70S6K signaling’ and ‘mTOR signaling’.

Another biological process whose genes were upregulated at D2 before expression levels drop off by D6 is the inflammatory response. This is exemplified by the TREM1 signaling pathway, which is the second most significant pathway enriched in genes upregulated at D2, and the fourth most downregulated at D6. TREM1 is a cell surface receptor that is expressed on neutrophils, monocytes, and macrophages. Upon activation by cellular pattern recognition receptors (PRRs), it promotes inflammation through activation of the transcription factors NF-κB and STAT3 [27]. DEGs that map to this pathway include *TREM1* itself and the transcription factor *STAT3*, cytokines *(IL1B*, *TNF*, *IL10*), extracellular (*TLR2*, *TLR4*) and intracellular (*TLR3*, *TLRs7-9*, *NOD1*) PRRs, and other intracellular signal transduction components (*MYD88*, *JAK2*, *LAT2*). Other pathways associated with innate immunity and inflammation were also identified. These include antigen presentation genes, from both class I (*PSMB8*, *PSMB9*, *SLA-1*, *SLA-3*, *TAP1*, *TAP2*, *TAPBP*) and class II pathways (*CII2A*, *SLA-DMA*, *SLA-DMB*, *SLA-DOA*, *SLA-DQB*, *SLA-DRA1*, and *SLA-DRB1*), and DEGs from the complement pathway (*C1R*, *C2*, *C4A*, *C3AR1*, *C5AR1*, *CD55*, *CFB*, *ITGAX*, and *SERPING1*).

The inflammatory response is a central component of innate immunity, and pro-inflammatory signaling is a prominent feature of the expression profile of PRRSV-infected gilts two days post-inoculation. Cells of the monocyte lineage, particularly monocytes and dendritic cells, have a central role in the recognition of pathogens and the instigation of innate immune responses. PRRSV has a tropism for CD163 positive monocyte cell lineages, and *in vitro* infection of this cell type in swine typically results in a suppression of pro-inflammatory signaling during the first 24 hours post-infection [28, 29]. *In vivo* however, the complex mixture of cell types and interactions between them lead to an inflammatory response being detected in multiple tissues from infected animals [24, 30, 31]. In fact, the strength of the inflammatory response to infection is an important distinguishing feature between highly pathogenic variants and more typical strains of PRRSV [30] and could contribute to differences in robustness to infection exhibited by nursery-stage animals [31].

**Fig 2:**
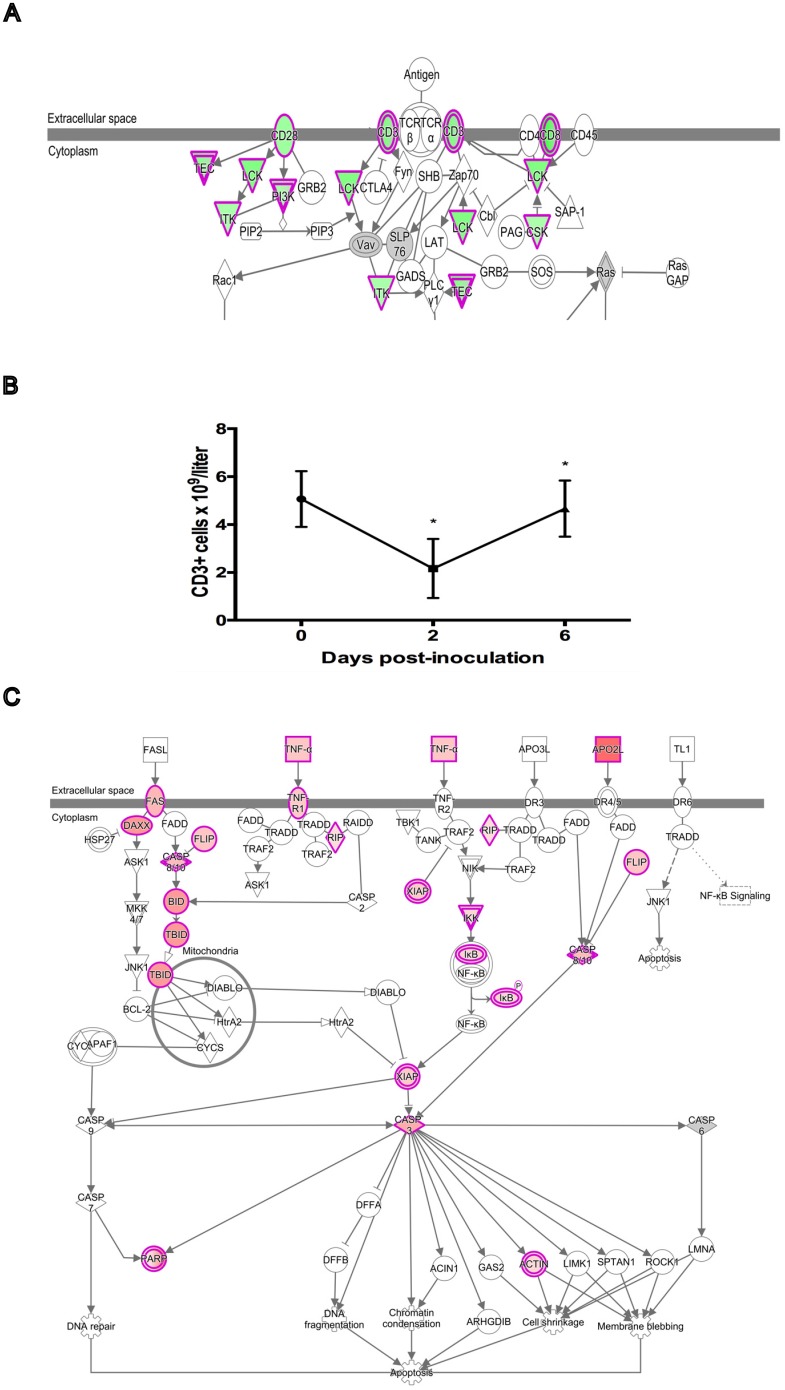
Relationship between Apoptosis and T Cell Gene Expression Two Days Post-inoculation with PRRSV. (A) T cell receptor signaling pathway from Ingenuity Pathway Analysis showing genes that are downregulated (green) on D2 compared to D0 in whole blood of PRRSV-inoculated gilts. Color intensity indicates magnitude of differential expression. (B) Plot of mean T cell (CD3+ve) number for the 16 experimental gilts during the first six days of infection showing the decline over the first two days of infection. Cell counts were obtained from a previously published dataset [10]. Asterisks indicate significant cell count differences between successive time-points (*P*<0.001). (C) Death receptor signaling pathway from Ingenuity Pathway Analysis showing genes that are upregulated (red) on D2 compared to D0. Color intensity indicates magnitude of differential expression. The data suggest that apoptosis is at least partly responsible for the decline in T cell numbers over the first two days of infection.

Many pro-apoptotic genes were also found to be upregulated at D2, mainly those associated with the extrinsic death receptor-signaling pathway. These include several death receptors, ligands, and associated signaling molecules (*TNF*, *TNFSF10*/*TRAIL*, *TNFRSF1A*, *FAS*, *DAXX*, *RIPK1*) and death effector caspases and their regulators (*CASP3*, *CASP10*, *CFLAR*). The upregulation of pro-apoptotic signaling at D2 corresponds with an acute drop in absolute leukocyte numbers, including T cells, and of T cell signaling in the blood of PRRSV-inoculated gilts at this time-point [10] (Fig 2). The T Cell Receptor (TCR) signaling and associated pathways such as ‘CTLA4 signaling in Cytotoxic T lymphocytes’ (CTL) and ‘iCOS-iCOSL signaling in T helper cells’ were downregulated at D2. DEGs that mapped to these pathways included T cell surface markers (*CD28*, *CD3D*, *CD3E*, *CD3G*, *CD8A*, and *CD8B*) and other intracellular signal transduction molecules (*BMX*, *CSK*, *ITK*, *LCK*, *MAP3K1*, and *PIK3CG*). Additional markers of cytolytic activity were also among the downregulated genes such as granzymes (*GZMK* and *GZMM*) and the pore forming protein PRF1.

Apoptotic cells have been observed in a number of tissues from PRRSV-infected pigs, including lung, lymph node, thymus, and reproductive tissues [32–35]. Interestingly, the majority of apoptotic cells in these tissues were not themselves infected with the virus, which suggests that PRRSV can indirectly induce apoptosis in uninfected, bystander cells. Recently, it was reported that PRRSV induced activation of the extrinsic apoptosis pathway in lymphocytes and monocytes of infected lymphoid organs [36]. The gene expression data in this paper support this finding, although these results require further validation by fluorescence-activated cell sorting in combination with a molecular assay to detect apoptotic cells. It is likely that one or a combination of cytokines activates the extrinsic apoptosis pathway, but the exact mechanism has yet to be determined. The genes of several cytokines that can trigger apoptosis in leukocytes were identified as upregulated at D2 in this study: these include IL1B, IL10, TNF, and TNFSF10/TRAIL. The latter two cytokines both signal through death receptors [37], and are therefore good candidates for mediating apoptosis via the extrinsic pathway in cells from infected gilts, but further research is needed to confirm this.

T cells constituted the largest leukocyte fraction among the PBMC of gilts in this experiment. They also exhibited the largest % reduction in cell count among all basic leukocyte types (T, B, NK, and monocytes) tested at D2 [10], which probably accounts for the observed decrease in T cell marker gene expression. The possible involvement of apoptosis in the reduction of total leukocyte counts has already been discussed. Apoptosis of lymphocytes in general has been observed in a variety of PRRSV-infected tissues [33–35]. More specifically, apoptosis of CD3 positive T cells and thymocytes has been observed in the lymph node, tonsil, and thymus of PRRSV-infected piglets [32, 38]. T cells are crucial to the development of an effective adaptive immune response against infection, so induction of apoptosis in these cells could contribute to the suppression of immune responses during PRRSV infection.

**Fig 3:**
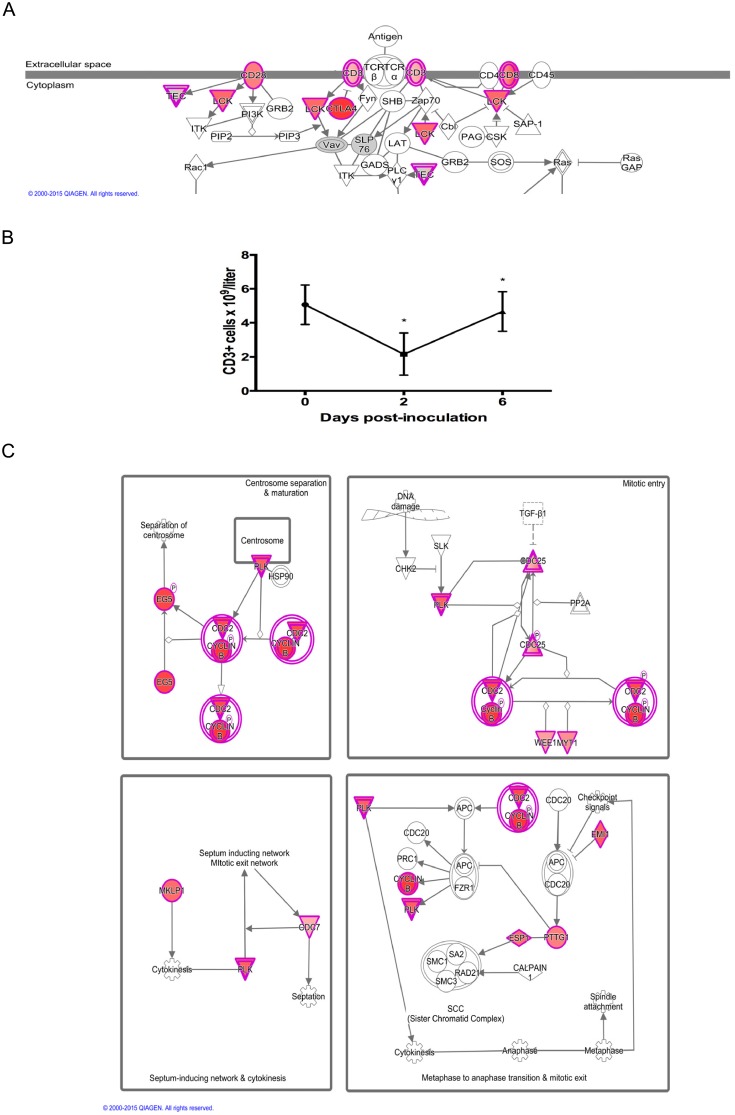
Relationship between T Cell and Mitosis Gene Expression Six Days Post-inoculation with PRRSV. (A) T cell receptor signaling pathway from Ingenuity Pathway Analysis showing genes that are upregulated (red) on D6 compared to D2 in whole blood of PRRSV-inoculated gilts. Color intensity indicates magnitude of differential expression. (B) Plot of mean T cell (CD3+ve) number for the 16 experimental gilts during the first six days of infection showing the rebound in T cell numbers between day 2 and day 6 post-infection. Cell counts were obtained from a previously published dataset [10]. Asterisks indicate significant cell count differences between successive time-points (*P*<0.001). (C) Mitosis signaling pathways from Ingenuity Pathway Analysis showing genes that are upregulated (red) on D6 compared to D2. Color intensity indicates magnitude of differential expression. The data suggest that mitosis is at least partly responsible for the rebound in T cell numbers between D2 and D6 post-inoculation.

At D6, the pattern of gene expression for apoptotic and T cell signaling was reversed from D2: apoptosis signaling was downregulated, while T cell signaling was upregulated. This was accompanied by an increase in T cell numbers, and increase in the expression of genes that feature in pathways associated with mitosis (Fig 3). These included ‘Mitotic roles of polo-like kinase’, ‘Cell cycle G2/M DNA damage checkpoint regulation’, and ‘Cell cycle control of chromosomal replication’. The top gene sets related to genes regulated by two transcription factors involved in cell cycle progression: E2F and MYC. The gene sets ‘DNA Repair’ and ‘Mitotic Spindle’ were also significant. Specific genes included cyclins (*CCNB1*, *CCNB2*, *CCNB3*), cell division regulatory kinases (*AURKA*, *CDC7*, *CDK1*, *CHEK1*, *PKMYT1*, *PLK1*, *PLK4*, *WEE1*) and phosphatases (*CDC25B*, *CDC25C*), DNA replication complex proteins (*CC45*, *CDC6*, *MCM2*, *MCM3*, *MCM4*, *ORC6*, *PCNA*, *RPA1*, *RPA3*) and chromosome separation proteins (*CENPE*, *CENPM*, *ESPL1*, *FBOX5*, *KIF11*, *KIF23*, *PTTG1*, *TOP2A*). This expression profile provided a clear indication of active cellular proliferation, as many of the mechanistic components of mitosis were upregulated. Many of the other most upregulated genes are known to be expressed in T cells such as cytolytic enzymes (*GZMA*, *GZMB*, *GZMK*), and cell surface proteins (*CD3D*, *CD3E*, *CD3G*, *CD8B*, *CTLA4*). We previously demonstrated that total leukocyte numbers rebounded in infected gilts at D6 post infection [10], and that the biggest % increase was in the T cell fraction. These expression results support this, and suggest that at least some of this rebound in the blood population is due to active cell proliferation, and not solely to an influx of leukocytes into the bloodstream from other sites. Other studies have also identified an increase in CD8B positive CTL in the peripheral blood of infected pigs at a similar time-point [39–41]. Whether this proliferation is caused by the activation of PRRSV-specific CTL or is a broader response of CTL to an immunostimulatory signal, such as polyclonal activation or cytokine signaling, is not clear. A role for IL10 in this process has previously been postulated [40], but we found that CTL proliferation and IL10 expression were negatively correlated in gilt whole blood. Irrespective of the cause, an expansion in CTL numbers does precede the eventual detection of IFN-γ by PRRSV-specific CTL from 2 weeks post-infection [40]. And although the appearance of this adaptive immune response is delayed and weaker than that of many viral infections, it does coincide with a drop in viremia that could indicate the importance of cell-mediated immunity for virus clearance [40].

### Blood gene expression indicators of severity of reproductive pathology in PRRSV-infected gilts

A comparison of whole blood expression profiles of LFM and HFM gilts was carried out for all 3 experimental time-points: D0, D2, and D6 post-infection. Tables of DEGs and gene sets whose expression is significantly positively or negatively correlated with the LFM phenotype are provided in supplementary files S3 and S4 Appendices respectively. For D0, 79 genes were upregulated in LFM gilts and 13 were downregulated. GSEA identified 12 gene sets whose expression were positively correlated with the LFM phenotype and 8 that were significantly negatively correlated. For D2, 568 DEGs were identified, 135 of which were upregulated in the LFM group, and 433 of which were downregulated. Ten gene sets were positively correlated with the LFM gene expression profile, while 22 had a negative correlation with LFM gene expression. For D6, 161 DEGs, 108 upregulated in LFM gilts and 53 downregulated, and 22 gene sets, 14 positively and 8 negatively correlated with the LFM phenotype, were identified.

Fifteen genes were consistently upregulated in LFM pig blood at all time-points (Table 1). Interestingly, at least seven of these genes (*CLU*, *GGT1*, *ITGA2B*, *ITGB3*, *PARVB*, *PF4*, and *TUBB1*) are known to be associated with platelet function. *PF4* (*CXCL4*) encodes a chemokine that is released from the alpha granules of activated platelets [42]. *ITGA2B* and *ITGB3* encode the heterodimeric glycoprotein components of the major plasma membrane integrin, which functions as a receptor for the clotting factors van Willebrand factor and fibrinogen in activated platelets, and initiates aggregation [43]. *PARVB* encodes affixin, part of a protein complex that associates with the major platelet integrin upon platelet activation and initiates reorganization of the cytoskeleton prior to platelet aggregation [44]. The transcript for *CLU* (clusterin), a complement lysis inhibitor, is one of the most abundant transcripts found in platelets [45]. The *GGT* gene encodes a platelet membrane-bound γ-glutamyltransferase enzyme that metabolizes extracellular glutathione [46]. Finally, *TUBB1* encodes a microtubule subunit that is expressed exclusively in platelets and megakaryocytes and is involved in platelet formation and release [47].

**Table 1:** Genes Upregulated in Gilts with Low Fetal Mortality at All Time-points.

Gene Symbol	Ensembl Gene ID	Log_2_ Fold Change D0	Log_2_ Fold Change D2	Log_2_ Fold Change D6
**CLU**	**ESSCG00000009668**	**0.75**	**0.75**	**0.68**
DOK5	ESSCG00000007488	0.54	0.56	0.89
**GGT1**	**ESSCG00000010056**	**0.47**	**0.43**	**0.42**
HEPH	ESSCG00000012363	0.49	0.43	0.58
HFM1	ESSCG00000006912	0.71	0.71	0.69
IQCD	ESSCG00000009877	0.77	0.58	0.58
**ITGA2B**	**ESSCG00000017357**	**0.58**	**0.47**	**0.77**
**ITGB3**	**ESSCG00000017306**	**0.77**	**0.48**	**0.71**
Novel Gene	ESSCG00000027611	0.55	0.44	0.58
**PARVB**	**ESSCG00000000024**	**0.33**	**0.40**	**0.50**
**PF4 (CXCL4)**	**ESSCG00000000243**	**1.25**	**0.74**	**0.81**
RHBDL2	ESSCG00000003651	0.65	0.52	0.66
**TUBB1**	**ESSCG00000007523**	**0.36**	**0.35**	**0.47**
ZNF529	ESSCG00000029875	0.41	0.39	0.38
ZNF674	ESSCG00000012264	0.30	0.33	0.35

Bold text indicates involvement in platelet function

Platelets are anucleated blood cells whose primary function is to effect hemostasis in response to vascular injury. Upon activation by collagen exposed from under the endothelial cell lining of blood vessels, platelets undergo a rapid conformational change that promotes platelet aggregation and the formation of a platelet plug at the wound site. They also release platelet chemotactic and clot-promoting factors into the plasma to accelerate hemostasis. Platelets have a secondary function as effector cells of inflammatory and innate immune responses. They are the earliest and most numerous cells to accumulate at sites of vascular infection, and share many of the innate immune functions of other myeloid leukocytes. They are capable of engulfing microbes [48], and many of the proteins released from activated platelets have either pro-inflammatory or antimicrobial functions. The previously mentioned PF4, for example, can directly kill the malaria parasite *Plasmodium falciparum* in infected erythrocytes [49]. Platelets can also facilitate CTL-mediated adaptive immune responses, as shown in mouse models of viral infections [50, 51]. A prior platelet deficiency, such as thrombocytopenia, can result in reduced survival rates or pathogen clearance in certain infections [51, 52]. One possibility is that the LFM gilts have a greater blood concentration of platelets than HFM gilts both prior to and during infection, which enhances the speed and strength of the initial innate, inflammatory response to PRRSV in the vasculature of the gilt uterine tissue. This could prevent or delay the establishment of a productive infection at this site. It could also prevent or limit further endothelial damage or downstream tissue damage resulting from hypoxic or inflammatory responses to endothelial damage.

**Fig 4:**
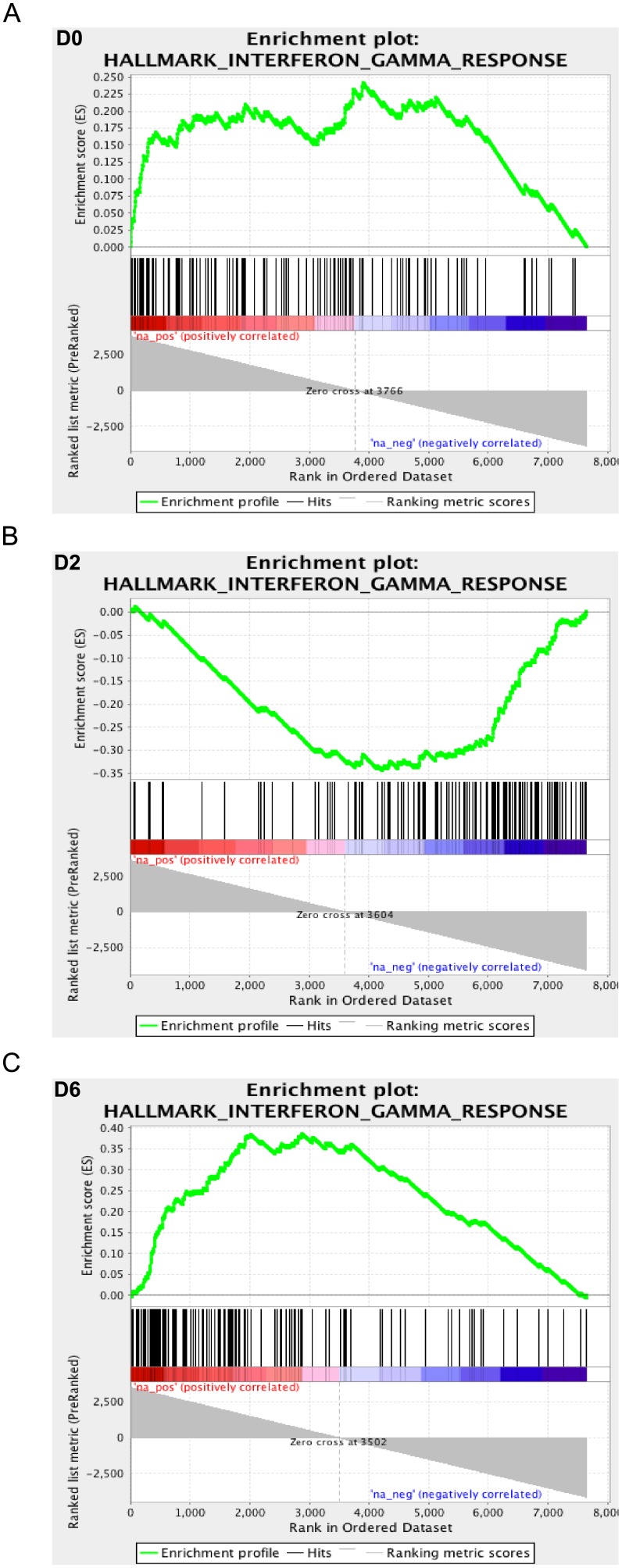
Temporal Expression Profile of Interferon-γ Signaling Gene Set in Gilts Exhibiting Low Fetal Mortality. Enrichment plots for the Interferon-γ signaling gene set produced by Gene Set Enrichment Analysis of transcriptional data from the blood of low and high fetal mortality (LFM and HFM) groups at three time-points during PRRSV-infection: D0 (A), D2 (B), and D6 (C). The enrichment score is calculated by walking down a list of genes ranked by their correlation with the LFM phenotype (green line), increasing a running-sum statistic when a gene in that gene set is encountered (each black vertical line underneath the enrichment plot) and decreasing it when a gene that isn’t in the gene set is encountered. The enrichment score is the maximum deviation from zero encountered in the walk. Magnitude and direction of correlation between expression of individual genes with LFM group is indicated on the color scale below the black lines with red indicating positive correlation and blue indicating negative correlation. Interferon-γ signaling gene expression was positively correlated with LFM (enriched in LFM) at D0 and D6 and negatively correlated with LFM on D2 (enriched in HFM).

Gene set enrichment analyses found that the expression of many of the significant gene sets oscillated between being positively or negatively correlated with the low fetal mortality across the three time-points. Interferon and pro-inflammatory signaling gene sets were among the most significant gene sets. Their expression was positively correlated with LFM phenotype at D0, negatively correlated at D2, and again positively correlated at D6 (Fig 4). These gene sets included ‘Interferon Alpha Response’, ‘Interferon Gamma Response’, ‘TNF-alpha signaling via NFKB’, and ‘Inflammatory Response’. Functional categories under these broader terms include interferon-inducible genes with antiviral function (*IFIT3*, *ISG15*, *ISG20*, *MX1*, *RSAD2*), cytokines and cytokine receptors (*CCL4*, *CXCL10*, *IL1A*, *IL10*, *S100A9*, *S100A12*, *TNFSF10*), PRRs (*NOD1*, *TLR2*, *TLR4*), NF-κB signaling genes (*CD14*, *MYD88*, *IL1A*, *NFKBIE*, *RELA*, *RELB*), and hemostasis (*GP1BA*, *ITGB3*, *PF4*, *SERPINB2*, *SERPINE1*). The discovery of elevated levels of these genes in LFM gilts prior to inoculation is particularly interesting, and this could contribute to the low fetal mortality phenotype. One interpretation of this result is that the LFM gilts were primed to respond more quickly and effectively to the initial stage of infection in the uterine vasculature and tissue. If this was the case, it offers the exciting prospect of identifying blood biomarkers that could be developed into a screening test to predict the extent of fetal pathology in gilts/sows prior to PRRSV infection. A similar discovery of innate immune gene expression prior to infection was recently made for a model of *Salmonella* infection in pigs [53]. In that study, the expression profiles of networks containing genes that had previously been implicated in resistance to *Salmonella* were positively correlated with a low shedding phenotype. Further research is warranted using larger numbers of biological replicates to confirm these findings, and to investigate the underlying reason for the elevated expression of these genes.

The lower level of pro-inflammatory and innate immune response gene signaling in LFM compared to HFM pigs at D2 also fits with the concept of a different dynamic in blood response to infection between the two groups. If the LFM pigs responded more quickly to infection, as inferred from the D0 expression profile, then by D2 pro-inflammatory signaling levels could have been returning to a lower level, whereas the slower responding HFM gilts were still in the initial phase of infection response. Again, these results mirror those seen in the *Salmonella* model where a similar difference in the dynamics of the immune response to infection in low and high shedders was observed between D0 and D2 [53]. At D6, one possibility for the switch back to higher levels of IFN-γ signaling in LFM than HFM gilt blood is attributable to the generation of this cytokine by PRRSV-specific activated T cells. One of the most upregulated genes is CLNK, an adapter protein that functions in TCR signaling whose expression is dependent on sustained IL2 stimulation [54]. It is also notable that the ‘IL2-STAT5 signaling’ gene set, that controls T cell proliferation, was significantly associated with LFM at D6, but not at D0.

**Fig 5:**
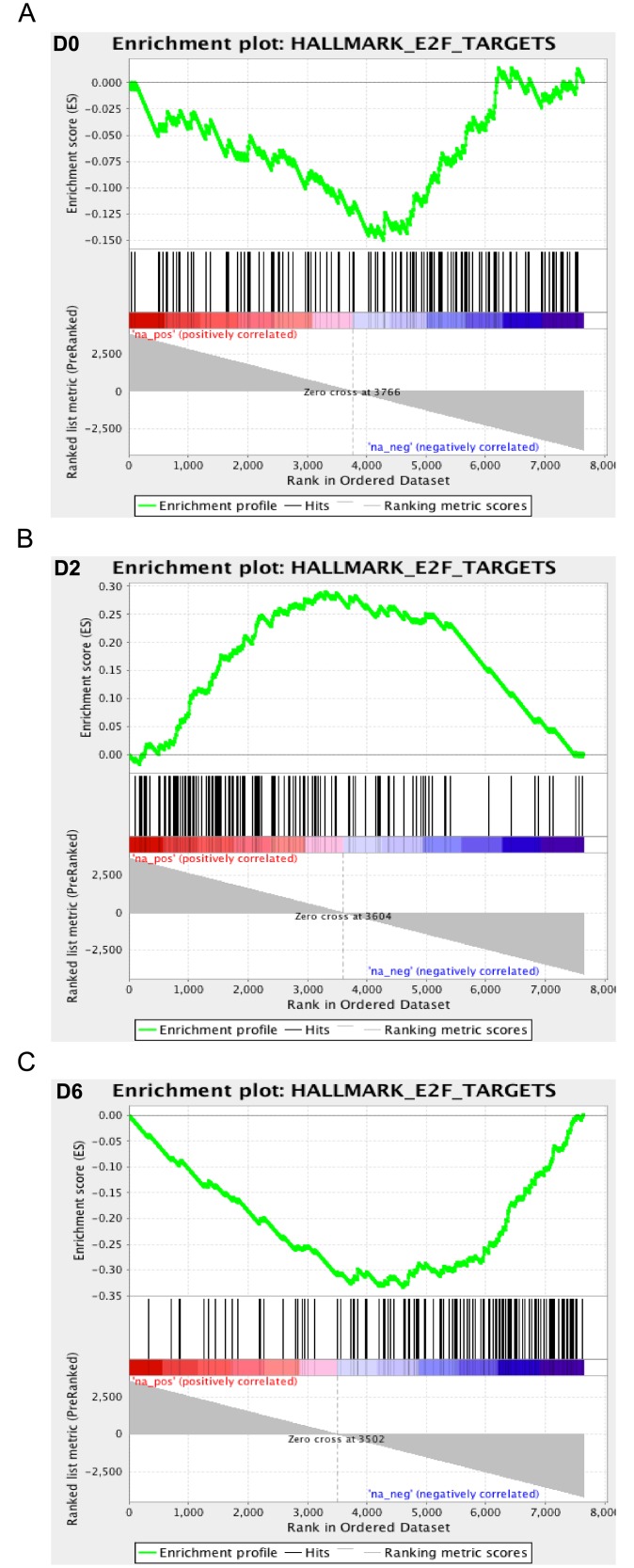
Temporal Expression Profile of E2F Target Gene Set in Gilts Exhibiting Low Fetal Mortality. Enrichment plots for the ‘E2F target’ gene set produced by Gene Set Enrichment Analysis of transcriptional data from the blood of low and high fetal mortality (LFM and HFM) groups at three time-points during PRRSV-infection: D0 (A), D2 (B), and D6 (C). E2F target gene expression is negatively correlated with LFM (enriched in HFM) at D0 and D6 and positively correlated with LFM on D2 (enriched in LFM).

Two other significant gene sets show the opposite oscillating pattern of gene expression to the interferon and inflammatory gene sets. Expression of the ‘MYC Targets v1’ and ‘E2F Targets’ gene sets are negatively correlated with LFM at D0 and D6, but positively correlated at D2 (Fig 5). Specific genes among these sets function in cell cycle signaling (*CCNB2*, *CCNE1*, *CDC25B*, *MYC*), DNA replication and recombination (*CDC45*, *PCNA*, *POLD2*, *RAD51AP1*), and cytokinesis (*AURKB*, *BUB3*, *CENPE*, *KIF22*). The negative relationship between interferon and cell cycle signaling at D0 and D6 may be attributable to the cellular effects of interferon signaling, while the positive correlation between MYC and E2F signaling at D2 and the LFM phenotype could relate to T cell activation. Among the 105 genes that were upregulated in LFM gilts at D2 were a number of genes associated with T lymphocytes and T cell receptor signaling. These genes include plasma membrane proteins (*CD3D*, *CD8B*, *CD27*, *CD28*, *CTLA4*, *ICOS*, *LCK*, *TRBV19*), cytolytic enzymes (*GZMA*, *GZMB*, *GZMK*), and intracellular signal transduction components (*LAT*, *LEF1*, *SH2D1A*). As discussed previously, there is a general decline in lymphocyte numbers in all gilts at D2 compared to D0. T cell numbers were numerically greater in LFM than HFM gilts at D2, but not statistically different. However, the expression profile of LFM gilts does indicate a greater extent of T cell activation at D2. Many of the most upregulated genes are known to be specifically upregulated following T cell activation. This includes three members of the CD28 family of T cell membrane proteins: *CD28*, *CTLA4*, and *ICOS*. These proteins bind to B7 family proteins on antigen presenting cells and transmit co-stimulatory signals to the T cell. Transcription of all three molecules is upregulated following initial TCR engagement with the MHC complex, particularly *CTLA4* and *ICOS*, which are not found on the surface of resting CTL or T helper cells [55, 56]. Likewise, expression of the cytolytic granzyme genes is controlled by TCR activation [57]. Again, these results fit with the hypothesis that LFM gilts are capable of initiating faster immune responses to infection, with a more rapid innate response succeeded by an earlier T cell response. Specific T cell responses are considered weak in PRRSV infections compared to many other viral pathogens, but they likely contribute to the eventual clearance of the virus and are an essential component of vaccines that are at least moderately effective at preventing infection.

Previously, using the complete set of PRRSV-inoculated gilts from the challenge experiment, we showed that absolute numbers of T helper cells during the first 6 days of infection reduced the odds of fetal death [6]. The positive association between T cell activation at D2 and fetal survival in this study supports a role for T cells in protecting against transplacental virus transmission. The relationship between gilt blood IFN-α response and fetal mortality rate appeared superficially to differ between the two analyses, with greater signaling at D0 being protective in this study and a risk factor for fetal death in the study of Ladinig *et al* [6]. Subtle differences in what was measured could be partly attributable. This study measured IFN-α pathway signaling at the gene level *in vivo*, whereas the negative correlation between IFN-α protein levels and fetal mortality in that study was obtained from *ex vivo* D0 blood cells following *in vitro* stimulation with PRRSV. In actuality, these assays were reporting on different properties of interferon signaling in cells from D0 blood. The measurement in whole blood in this study quantified basal differences in interferon pathways between LFM and HFM gilts prior to exposure to PRRSV, whereas results of the PBMC stimulation assay reported by Ladinig *et al*. [6] detected differences in the early response to the virus in cells that had not previously been exposed to PRRSV. It could be that a higher basal level of interferon signaling prior to infection results in a protective surge in innate immunity of short duration, whereas a slower and more prolonged activation of this pathway in response to *de novo* exposure to PRRSV is ultimately detrimental to fetal life. Interestingly, the cumulative levels of interferon-alpha released by *ex vivo* PRRSV-stimulated blood cells over a 19-day period were positively associated with odds of fetal death in the same study [6].

One limitation of this study is that it did not also profile gene expression at the maternal-fetal interface or in the fetal compartment of the reproductive tract of these gilts. Events at these sites are likely the most critically important in determining disease outcome for the fetus. PRRSV RNA in both the uterine endometrium and the fetus itself are very strong predictors of fetal mortality [6], and it has been postulated that PRRSV-induced damage to the placenta and its attachment to uterine epithelium are the primary cause of fetal death in reproductive PRRS [58]. Although the expression of multiple sets of immune genes in LFM gilt blood was associated with low viral load in the reproductive tract, this was not the case for other tissues tested, including for blood itself. Further research is required to determine how the detected changes in immune gene expression specifically contribute to the control of PRRSV replication in the uterus, but not in other gilt tissues.

In conclusion, these analyses have revealed for the first time detailed gene expression networks that underpin the early response to type 2 PRRSV infection in the blood of pregnant gilts. In particular, we provide evidence that PRRSV-induced apoptosis of bystander cells likely contributes to the leukopenia detected two days post-infection, and that the rebound in cell numbers observed by day 6 is at least partly due to leukocyte cell proliferation. Additionally, this study has found whole blood expression signatures in pregnant females that are associated with subsequent low levels of reproductive pathology. Gilts with low fetal mortality had a greater basal expression of platelet, interferon pathway and other innate immune genes prior to infection that lead to a more rapid progression to an adaptive immune response. Although more work is needed to link these expression patterns to a mechanistic model of protection against transplacental virus transmission, these results could be the starting point for the development of biomarkers to select for gilts that would be likely to be more resilient to reproductive pathology in the event of gestational PRRSV infection.

## Supporting Information

S1 Appendix Time-course Differentially Expressed Gene Lists. Excel file of lists of differentially expressed genes across days for all 16 experimental gilts. 5 tabs.
(XLSX)

S2 Appendix Significant Pathways from the Time-course Analysis. Excel file of lists of significant pathways enriched for differentially expressed genes across days (all 16 experimental gilts). 4 tabs.
(XLSX)

S3 Appendix Differentially Expressed Genes between Fetal Mortality Groups. Excel file of lists of whole blood differentially expressed genes for comparison of low and high fetal mortality groups. 10 tabs.
(XLSX)

S4 Appendix Significant Gene Sets from the Fetal Mortality Group Analysis. Excel file of lists of gene sets whose expression positively or negatively correlates with low fetal mortality. 3 tabs.
(XLSX)

S1 Fig Selection of Low and High Fetal Mortality Groups. Scatter plot of % dead fetuses against mean PRRSV RNA concentration (log_10_ copies/mg) in fetal thymus for all litters from PRRSV-challenged gilts. Gilts selected for the low fetal mortality group (LFM, green circles) and high fetal mortality group (HFM, red squares) are shown together with non-selected gilts (NS, black triangles). Dashed lines indicate mean values.
(PDF)
