# Supplementary figures and images for: Differences in Whole Blood Gene Expression Associated with Infection Time-Course and Extent of Fetal Mortality in a Reproductive Model of Type 2 Porcine Reproductive and Respiratory Syndrome Virus (PRRSV) Infection
Source: PLoS One. 2016 Apr 19;11(4):e0153615. doi: 10.1371/journal.pone.0153615 (PMC4836665; doi:10.1371/journal.pone.0153615)

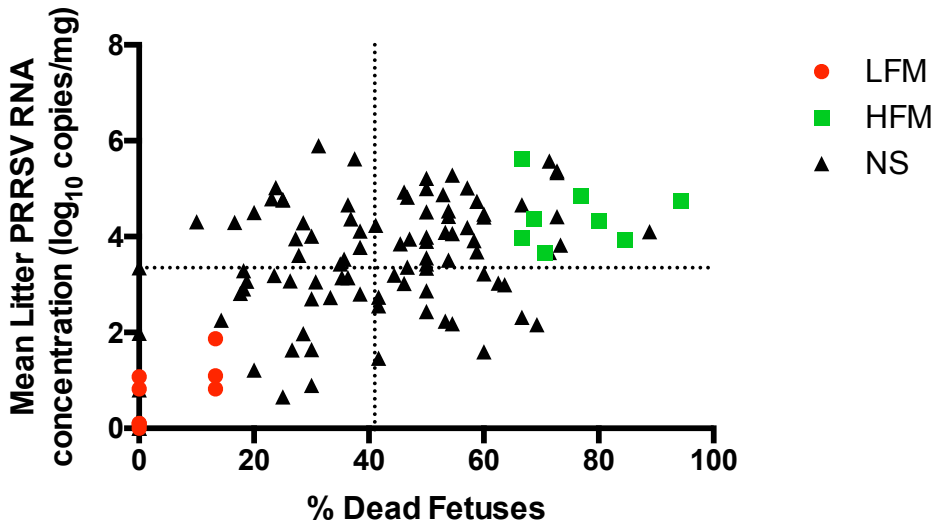

Supplement: S1 Fig — Scatter plot of % dead fetuses against mean PRRSV RNA concentration (log10 copies/mg) in fetal thymus for all litters from PRRSV-challenged gilts. Gilts selected for the low fetal mortality group (LFM, green circles) and high fetal mortality group (HFM, red squares) are shown together with non-selected gilts (NS, black triangles). Dashed lines indicate mean values. (PDF) [file pone.0153615.s005.pdf]
